# Establishment and validation of a simple nomogram for predicting early postpartum stress urinary incontinence among women with vaginal delivery: a retrospective study

**DOI:** 10.1186/s12905-023-02160-2

**Published:** 2023-01-09

**Authors:** Chuangchuang Xu, Ying Guo, Xiaolei Chi, Yiyao Chen, Lei Chu, Xinliang Chen

**Affiliations:** 1 Department of Obstetrics and Gynecology, International Peace Maternity and Child Health Hospital, School of Medicine, Shanghai Jiao Tong University, Shanghai, 200030 China; 2 Shanghai Key Laboratory of Embryo Original Disease, Shanghai, 200030 China; 3 Shanghai Municipal Key Clinical Specialty of Gynecologic Oncology, Shanghai, 200030 China

**Keywords:** Vaginal delivery, Stress urinary incontinence (SUI), Nomogram, Early postpartum period

## Abstract

**Background:**

Stress urinary incontinence (SUI) is a common public health issue that negatively impacts the quality of life for women worldwide, of which early detection and rehabilitation are consequently pivotal. The aim of this study is to establish a simple nomogram for identifying women at risk of postpartum SUI.

**Methods:**

A retrospective study was conducted in a tertiary specialized hospital in Shanghai, China. The study included only women with singleton, full-term, and vaginal deliveries. 2,441 women who delivered from July 2019 to November 2019 were included in the training cohort, and 610 women who delivered from January 2022 to February 2022 were included in the validation cohort. SUI was determined by the International Consultation on Incontinence Questionnaire-Urinary Incontinence Short Form (ICIQ-UI-SF). Univariate and multifactorial logistical regression were used to identify independent risk factors for postpartum SUI and further construct the nomogram accordingly. Based on concordance statistics (C-statistics), calibration curves, and decision curve analyses, we evaluated the performance of the nomogram in the training cohort and the validation cohort. In addition, the model was validated internally in the training cohort through cross-validation.

**Results:**

There were no significant statistically differences in important baseline data such as age, pre-pregnancy BMI, and parity between the training and validation cohorts. SUI was observed in 431 (17.6%) and 125 (20.5%) women in the training and validation cohorts, respectively. According to the regression analysis, age, parity, second stage of labor, infant weight, and forceps delivery were included in the nomogram. The nomogram had a C-statistic of 0.80 (95% confidence interval [CI] 0.74–0.85) for predicting SUI. C-statistics were stable in both internally cross-validated training cohort (mean 0.81) and validation cohort (0.83 [95% CI 0.79–0.87]). The nomogram’s calibration curve was near the ideal diagonal line. Additionally, the model exhibited a positive net benefit from the decision curve analysis.

**Conclusion:**

We have created a nomogram that can be utilized to quantify the risk of postpartum SUI for women with vaginal delivery. The model might contribute to predicting early postpartum SUI, thereby facilitating the management of SUI.

**Supplementary Information:**

The online version contains supplementary material available at 10.1186/s12905-023-02160-2.

## Introduction

Stress urinary incontinence (SUI), which is defined as the involuntary loss of urine on effort or physical exertion or on sneezing or coughing, is one of the most common patterns of UI [1]. The prevalence of SUI was reported to vary from 15.1 to 41.7% [2–4]. In addition, UI could persist to 12 years in about three-quarters of women who had reported UI at 3 months [5], showing 24% and 37.9% prevalence of persistent UI at 6 and 12 years postpartum respectively [6]. Women with persistent UI had lower quality of life[5,7,8]and huge social costs burden [9, 10]. Therefore, early identification of postpartum SUI will be beneficial.

Although recent advances have been made in the last few decades, the factors and mechanisms underlying SUI remain unknown. The risk factors of SUI, which have been mostly reported, included delivery mode, parity, maternal age, and body mass index (BMI) [5, 11, 12]. The risk of SUI was higher among women who had vaginal deliveries [13]. Forceps delivery was reported to increase the risk of long-term SUI compared with cesarean delivery [13–15]. Older age at first birth, greater parity, and overweight/obesity were previously found to be associated with persistent UI [5, 10, 16, 17]. Moreover, a higher BMI was demonstrated to be correlated with more severe UI symptoms [18]. In addition, birthweight of the baby [19–21], not using oxytocin [2, 22], low income, high education, living in a rural area, and physical work during pregnancy were also found to be risk factors for maternal SUI [2]. However, the role of maternal and obstetrical indicators in helping to identify SUI remains unknown in women with vaginal delivery.

Herein, a series of maternal and obstetrical characteristics were investigated for detection of SUI. We aim to develop a nomogram for predicting the risk of SUI in women with vaginal delivery, which will facilitate the management of SUI and confer great clinical value. We hypothesize that the nomogram, which is based on maternal and obstetrical characteristics could quantify the risk of developing SUI.

## Methods

### Study design and population

This single-center observational study was done at the International Peace Maternity and Child Health Hospital (IPMCH), Shanghai Jiao Tong University School of Medicine, designated for early postpartum women with vaginal delivery. The protocol was approved by the Ethics Committee of the IPMCH (No. 2016-55), and the requirement for individual consent was waived.

Women who delivered from July 2019 to November 2019 and women who delivered from January 2022 to February 2022 were included in the training cohort and the validation cohort, respectively. We enrolled women who met the following criteria: (a). Singleton, cephalic, and full-term delivery; (b). 42–100 days postpartum. Following were the exclusion standards: (a). History of cesarean delivery (women with any prior history of cesarean delivery in their lifetime were excluded, and those who underwent a successful vaginal birth after cesarean were also excluded from the study) or miscarriage after 20 gestational weeks; (b). Abnormal postpartum recovery (including vaginal bleeding, failure of the uterus to contract into the pelvis, poor healing of a perineal laceration or lateral episiotomy wound, and abnormal leukorrhea); (c). Incomplete records (e.g., height, weight, and labor summaries, etc.).

### Data collection and definition

All data were obtained from electronic medical record (EMR) and electronic health record (EHR). General demographic characteristics included pre-pregnancy BMI, maternal age, educational level, gravidity, and parity; Baseline characteristics during pregnancy included weight gain in pregnancy, diabetes (gestational/pregestational), hypertensive disorders, other complications (defined as anemia, impaired liver and kidney function, and abnormal thyroid function), gestational age, and infant weight; Baseline characteristics during labor included induction of labor (including oxytocin, prostaglandin, and cervix balloon mechanical induction of labor), epidural anesthesia, second stage of labor (time from cervix fully dilated to complete delivery of fetus), episiotomy (routine episiotomies are mediolateral), perineal lacerations, and instrumental delivery, namely forceps delivery. During the period, only 5 women were found to deliver assisted by vacuum. However, the 5 women were excluded due to meeting the exclusion standards. Thus, vacuum-assisted delivery has not been included.

### Study outcomes

The primary outcome of this study was SUI, which was defined by the International Urogynecological Association (IUGA) and the International Continence Society (ICS) as a complaint of involuntary loss of urine on effort or physical exertion (e.g., sporting activities), or on sneezing or coughing [1]. Women were further assessed by a physician using the International Consultation on Incontinence Questionnaire Short Form (ICIQ-UI-SF) [23] if they self-reported symptoms of urine leaking after delivery. Three rated questions and one non-rated question make up the ICIQ-UI-SF. The rated questions are as follows: How much urine do you usually leak? How often do you leak urine? How much does leaking urine impact your daily life? With a minimum score of 0 and a maximum score of 21, the combined score for the three questions was recorded. Cut-off scores were established at 0 (no incontinence) and ≥ 1 (UI). The type of UI was determined primarily by non-rated question on the questionnaire. SUI was diagnosed in women who chose “leaks when you cough or sneeze” or “leaks while you are physically active or exercising” from the list of options. UUI was diagnosed in women who chose "leaks before you can get to the toilet” or “leaks when you are asleep” or “leaks when you have finished urinating and are dressed” from the list of options. Women were diagnosed with MUI when they had symptoms of both SUI and UUI. This questionnaire is now available in Chinese, and its validity and accuracy have been well validated [24].

### Statistical analysis

The frequency (percentage) of categorical variables and the median (interquartile range) or mean (standard deviation) of continuous variables were used to report descriptive statistics. Mann–Whitney U test and the χ^2^ test were used to evaluate differences between medians or means and between proportions, respectively.

In the entire training cohort, univariable analysis was utilized to pinpoint significant factors connected to SUI. In multivariable logistic regression models, variables having a univariable link to SUI (*P* < 0.2) were added, and backwards stepwise selection was carried out with an improvement in goodness of fit measured by a decrease in the Akaike information criterion. A nomogram for SUI likelihood was developed based on the findings of the final regression analysis.

Concordance statistics (C-statistic) and 95% confidence intervals (CI) were computed to evaluate the nomogram model's capability to distinguish patients who will suffer from SUI. Furthermore, the C-statistics between the nomogram and each independent predictor were compared using the Delong test. Calibration curves were developed by bootstraps of 1000 resamples to analyze the agreement between nomogram predictions and actual observations in the training cohort. Decision curve analysis was performed by estimating the net benefits at various threshold probabilities of SUI to evaluate the clinical utility of the predictive nomogram.

Internal validation of the model's stability was carried out via cross-validation, which involved randomly dividing the training cohort's patients into ten equal samples. To create logistic regression models, nine of these samples were used, and the final sample was then given the model coefficients. The mean C-statistic for iteration was computed after this procedure was repeated ten times. Additionally, the model was applied to a validation dataset and evaluated using the C-statistic, calibration, and decision curve analysis to evaluate its external validity.

Statistical and graphing software were done with R version 4.1.3. All statistics were two–sided tests, and *P* < 0.05 was considered statistically significant.

## Results

### General characteristics

**Fig. 1:**
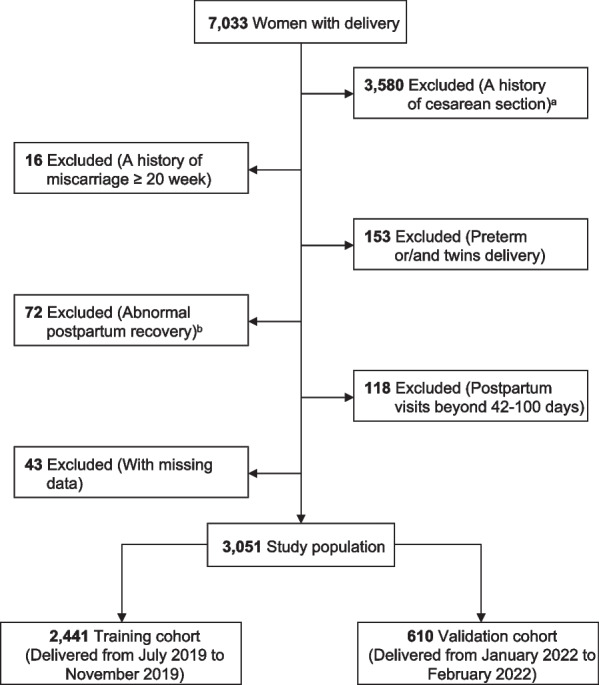
Flow chart for identification of eligible study population. ^a^Women with any prior history of cesarean delivery in their lifetime were excluded, and those who underwent a successful vaginal birth after a cesarean were also excluded from the study. ^b^This includes vaginal bleeding, failure of the uterus to contract into the pelvis, poor healing of a perineal laceration or lateral episiotomy wound, and abnormal leukorrhea

A total of 7033 women delivered during the study period, and 3,982 women were excluded as follows: 3580 had a history of cesarean delivery; 153 had a history of preterm or/and twin delivery; 16 had a history of miscarriage after 20 gestational weeks; 118 conducted postpartum visits beyond 42–100 days; 72 had abnormal postpartum recovery; and 43 had missing baseline data. Finally, 3,051 women were enrolled in this study, with 2,441 entering the training cohort and 610 entering the validation cohort (Fig. 1). There were no statistically significant differences in age (31 [28, 32] vs. 31 [28, 32], *P* = 0.677), pre-pregnancy BMI (22.3 [19.8, 26.2] vs. 22.3 [19.8, 26.3], *P* = 0.674), and proportion of parity ≥ 2 (975 [39.9] vs. 223 [36.6], *P* = 0.138) between the training and validation cohorts (Table 1). In addition, the difference in age (31 [28, 32] vs. 31 [28, 32], *P* = 0.928), pre-pregnancy BMI (22.4 [19.8, 26.3] vs. 22.3 [19.8, 26.2], *P* = 0.873), and proportion of parity ≥ 2 (1542 [38.7] vs. 1198 [39.3], *P* = 0.662) between the excluded and included populations was not statistically significant (Additional file 1: sTable 1). SUI occurred in 556 (18.2%) of cases overall, with 431 (17.6%) and 125 (20.5%) in the training cohort and validation cohort, respectively (Table 1). The ICIQ-UI-SF scores for the training and validation cohorts were 0 (0, 4.0) and 0 (0, 4.5).

**Table 1:** Demographics, pregnancy and delivery characteristics of training cohort and validation cohort, respectively

Variables	Training cohort, M (P25, P75)/N (%)				Validation cohort, M (P25, P75)/N (%)				
	Overall (N = 2441)	Non-SUI (N = 2010)	SUI (N = 431)	P* value	Overall (N = 610)	Non-SUI (N = 485)	SUI (N = 125)	P* value	*P*† value
Age, y	31 (29, 33)	30 (28, 33)	33 (31, 36)	< 0.001	31 (29, 33)	30 (28, 33)	33 (30, 36)	< 0.001	0.677
Pre-pregnancy BMI, kg/m^2^	22.3 (19.8, 26.2)	21.7 (19.6, 25.5)	25.1 (22.4, 29.2)	< 0.001	22.3 (19.8, 26.3)	21.8 (19.5, 25.8)	24.6 (21.7, 29.0)	< 0.001	0.674
*Education*				0.606				0.928	0.746
High school or low	72 (2.9)	62 (3.1)	10 (2.3)		21 (3.4)	16 (3.3)	5 (4.0)		
Junior college or university	1680 (68.8)	1377 (68.5)	303 (70.3)		423 (69.3)	337 (69.5)	86 (68.8)		
Graduate or above	689 (28.2)	571 (28.4)	118 (27.4)		166 (27.2)	132 (27.2)	34 (27.2)		
*Gravidity*				0.896				0.323	0.971
1	752 (30.8)	618 (30.7)	134 (31.1)		185 (30.3)	170 (35.1)	35 (28.0)		
2	799 (32.7)	662 (32.9)	137 (31.8)		202 (33.1)	158 (32.6)	44 (35.2)		
≥ 3	890 (36.5)	730 (36.3)	160 (37.1)		223 (36.6)	157 (32.4)	46 (36.8)		
*Parity*				< 0.001				< 0.001	0.138
1	1466 (60.1)	1369 (68.1)	97 (22.5)		387 (63.4)	350 (72.2)	37 (29.6)		
≥ 2	975 (39.9)	641 (31.9)	334 (77.5)		223 (36.6)	135 (27.8)	88 (70.4)		
Weight gain in pregnancy, kg	13.0 (10.9, 16.0)	13.1 (10.9, 16.0)	13.0 (11.0, 16.0)	0.681	13.4 (11.0, 16.0)	13.3 (11.0, 16.0)	13.8 (10.9, 16.0)	0.937	0.491
*Diabetes (gestational/pregestational)*				0.160				0.251	0.484
No	2110 (86.4)	1747 (86.9)	363 (84.2)		520 (85.2)	418 (86.2)	102 (81.6)		
Yes	331 (13.6)	263 (13.1)	68 (15.8)		90 (14.8)	67 (13.8)	23 (18.4)		
*Hypertensive disorders*				0.185				0.099	0.674
No	2310 (94.6)	1896 (94.3)	414 (96.1)		574 (94.1)	452 (93.2)	122 (97.6)		
Yes	131 (5.4)	114 (5.7)	17 (3.9)		36 (5.9)	33 (6.8)	3 (2.4)		
*Other complications*				0.316				0.237	0.59
No	2209 (90.5)	1825 (90.8)	384 (89.1)		547 (89.7)	439 (90.5)	108 (86.4)		
Yes	232 (9.5)	185 (9.2)	47 (10.9)		63 (10.3)	46 (9.5)	17 (13.6)		
Gestational age, week	39 (38, 40)	39 (38, 40)	39 (38, 40)	< 0.001	39 (38, 40)	39 (38, 40)	39 (38, 40)	0.209	0.103
Infant weight, g	3330 (3090, 3575)	3290 (3050, 3520)	3515 (3290, 3820)	< 0.001	3310 (3086, 3578)	3280 (3045, 3525)	3490 (3220, 3725)	< 0.001	0.702
*Induction of labor*				0.622				0.935	0.957
No	1546 (63.3)	1278 (63.6)	268 (62.2)		385 (63.1)	307 (63.3)	78 (62.4)		
Yes	895 (36.7)	732 (36.4)	163 (37.8)		225 (36.9)	178 (36.7)	47 (37.6)		
*Epidural anesthesia*				< 0.001				0.008	0.125
No	1091 (44.7)	847 (42.1)	244 (56.6)		251 (41.1)	186 (38.4)	65 (52.0)		
Yes	1350 (55.3)	1163 (57.9)	187 (43.4)		359 (58.9)	299 (61.6)	60 (48.0)		
Second stage of labor, min	35 (24, 57)	31 (22, 53)	53 (37, 112)	< 0.001	37(23, 58)	32(22, 54)	50 (35, 90)	< 0.001	0.806
*Forceps delivery*									0.048
No	2262 (92.7)	879 (93.5)	383 (88.9)	0.001	550 (90.2)	448 (92.4)	102 (81.6)	0.001	
Yes	179 (7.3)	131 (6.5)	48 (11.1)		60 (9.8)	37 (7.6)	23 (18.4)		
*Episiotomy*				0.128				0.246	0.303
No	1984 (81.3)	1622 (80.7)	362 (84.0)		484 (79.3)	390 (80.4)	94 (75.2)		
Yes	457 (18.7)	388 (19.3)	69 (16.0)		126 (20.7)	95 (19.6)	31 (24.8)		
*Perineal lacerations*				0.026				0.007	0.426
None	609 (24.9)	500 (24.9)	109 (25.3)		156 (25.6)	113 (23.3)	43 (34.4)		
I	1089 (44.6)	876 (43.6)	213 (49.4)		255 (41.8)	201 (41.4)	54 (43.2)		
II and above	743 (30.4)	634 (31.5)	109 (25.3)		199 (32.6)	171 (35.3)	28 (22.4)		

BMI, body max index; SUI, stress urinary incontinence

*P value for difference between women with SUI versus non-SUI

^†^*P* value for training cohort versus validation cohort for overall characteristic

### Selected factors for model

After univariable analysis, age, pre-pregnancy BMI, parity, diabetes, hypertensive disorders, gestational age, infant weight, epidural anesthesia, second stage of labor, forceps delivery, episiotomy, and perineal lacerations were entered into the multivariable logistic regression analysis. The multivariable analyses demonstrated that the occurrence of SUI was significantly correlated with second stage of labor, parity, age, and forceps delivery (*P* < 0.001); however, pre-pregnancy BMI, diabetes, hypertensive disorders, gestational age, infant weight, epidural anesthesia, episiotomy, and perineal lacerations were not associated with SUI. Due to the good log-likelihood ratio achieved and concordance index obtained via step-down selection, infant weight (*P* = 0.055) was also included in the final model (Table 2).

**Table 2:** Multivariable analysis of the training cohort

Variables	Multivariate analysis		Selected factors for model	
	OR (95% CI)	*P* value	OR (95% CI)	*P* value
Age	1.17 (1.09–1.25)	< 0.001	1.14 (1.09–1.19)	< 0.001
Pre-pregnancy BMI, kg/m^2^	0.97 (0.92–1.03)	0.371		
*Parity*				
1	1 [Reference]			
≥ 2	5.95 (4.07–8.80)	< 0.001	5.60 (4.07–7.79)	< 0.001
*Diabetes (gestational/pregestational)*				
No	1 [Reference]			
Yes	0.97 (0.68–1.37)	0.862		
*Hypertensive disorders*				
No	1 [Reference]			
Yes	0.84 (0.45–1.48)	0.561		
Gestational age, week	0.98 (0.86–1.12)	0.787		
Infant weight, g	1.00 (1.00–1.00)	0.141	1.00 (1.00–1.00)	0.055
*Epidural anesthesia*				
No	1 [Reference]			
Yes	1.16 (0.88–1.53)	0.297		
Second stage of labor, min	1.02 (1.01–1.03)	< 0.001	1.02 (1.01–1.03)	< 0.001
*Forceps delivery*				
No	1 [Reference]			
Yes	6.41 (3.53–11.88)	< 0.001	6.08 (3.85–9.56)	< 0.001
*Episiotomy*				
No	1 [Reference]			
Yes	0.84 (0.43–1.60)	0.591		
*Perineal lacerations*				
None	1 [Reference]			
I	0.80 (0.52–1.26)	0.327		
II and above	0.94 (0.57–1.57)	0.816		

BMI, body max index; OR, odds ratio; CI, confidence interval

### Risk prediction nomogram establishment

**Fig. 2:**
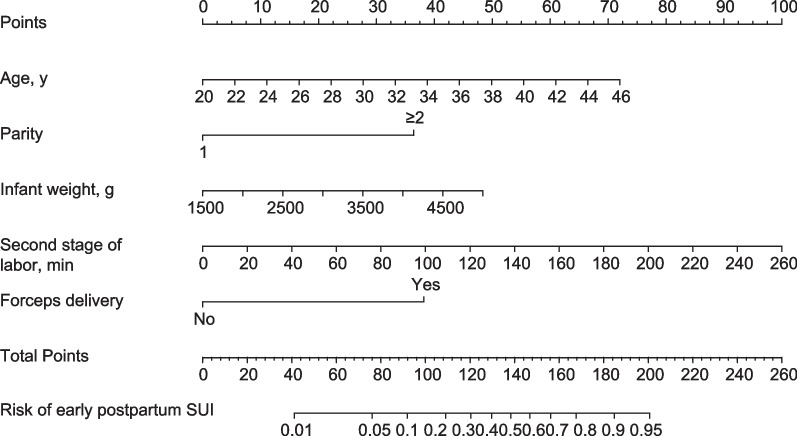
A nomogram predicting the early postpartum urinary incontinence (SUI) for women with vaginal delivery. On the axis of the point scale, the value of each variable was assigned a score. The probability of early postpartum SUI might be determined by adding up each individual score, and by projecting the result to the lower total point scale

The final regression analysis was used to create a nomogram for predicting SUI. Age, parity, infant weight, second stage of labor, and forceps delivery were used to obtain a total score. Each of these variables' values received a score on the axis of a point scale. Each individual score could be readily added up to create a total score, and by extrapolating the total score to the entire point scale, the likelihood of SUI could be calculated (Fig. 2).

### Performance of the nomogram

**Fig. 3:**
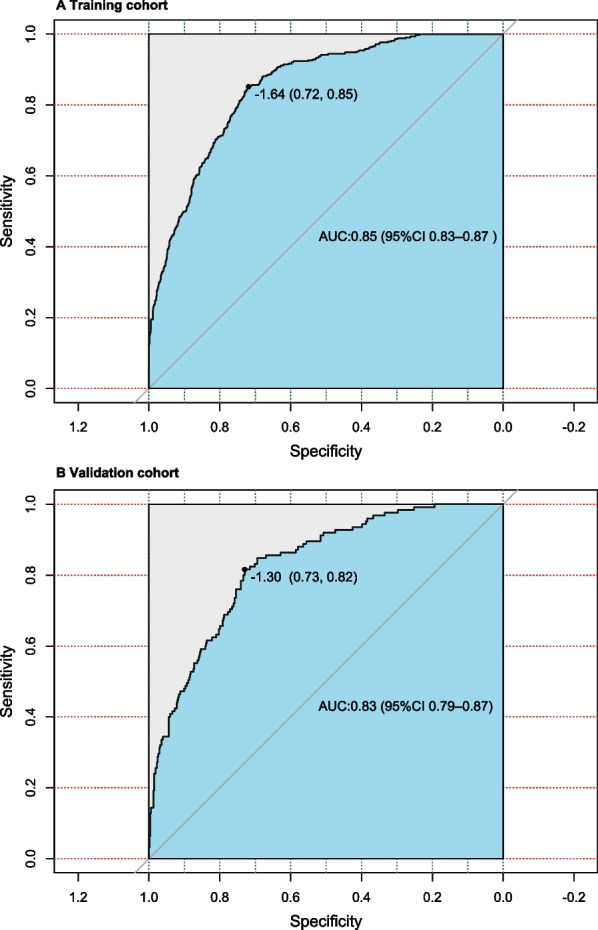
Receiver operating characteristic (ROC) curve. Abbreviations: AUC, area under the ROC curve, equal to C-statistic value

**Fig. 4:**
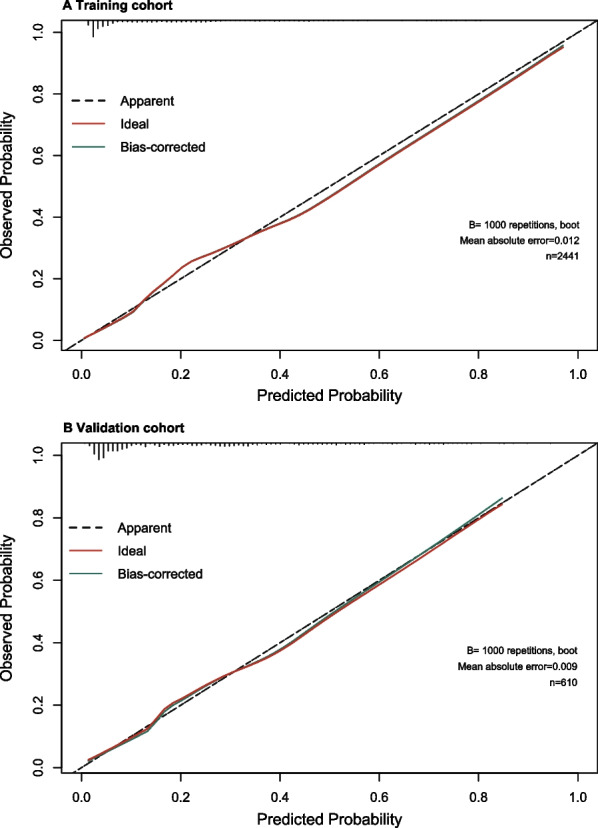
Calibration curves for the nomogram

**Fig. 5:**
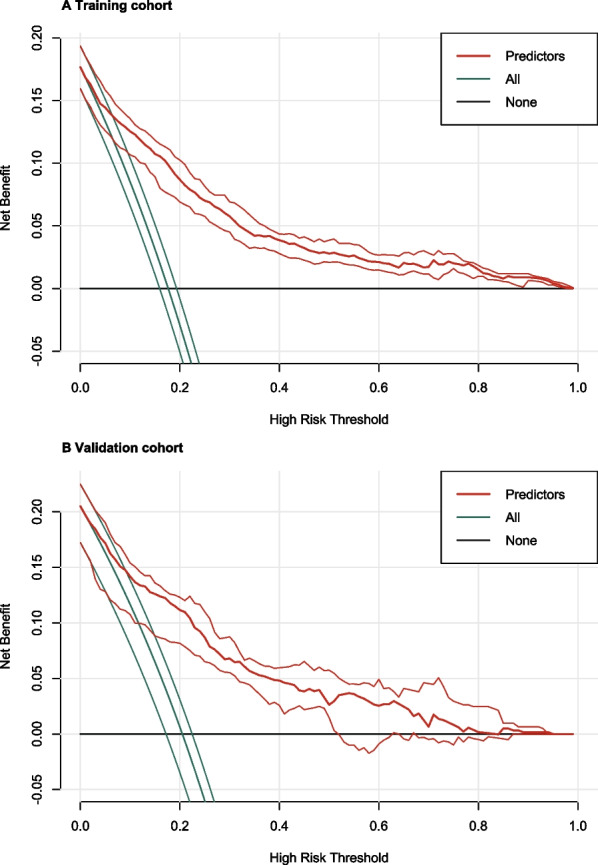
Decision curve analyses demonstrating the net benefit associated with the use of the nomogram on the detection of early postpartum stress urinary incontinence (SUI)

Using C-statistics, we evaluated the nomogram's discriminatory power toward women with SUI. The C-statistic for the nomogram used to predict SUI in the training cohort was 0.85 (95% CI 0.83–0.87) (Fig. 3). Both internally cross-validated training cohort (mean 0.81) and validation cohort (0.83 [95% CI 0.79–0.87]) showed stable C-statistic values (Fig. 3). The ability to predict SUI incidence was compared using the Delong test of the receiver operating characteristic (ROC) analysis. The nomogram's C-statistic was clearly superior to any one of the independent factors alone (both *P* < 0.001) (Table 3). In the training and validation cohorts, a calibration curve overlapped the ideal line, demonstrating good agreement between the actual probabilities and the SUI probabilities predicted by the nomogram. (Fig. 4). In the training cohort and the validation cohort, the threshold probabilities for the positive net benefit associated with using the nomogram to detect SUI varied from 0.00 to 0.99 and 0.00 to 0.94, respectively (Fig. 5).

**Table 3:** C-statistics for the nomogram and model variables in the training and validation cohorts

Variables	Training cohort		Validation cohort	
	C-statistic (95% CI)	P* value	C-statistic (95% CI)	P* value
Nomogram	0.85 (0.83–0.87)		0.83 (0.79–0.87)	
Age	0.74 (0.71–0.76)	< 0.001	0.70 (0.64–0.75)	< 0.001
Parity	0.73 (0.71–0.75)	< 0.001	0.71 (0.67–0.76)	< 0.001
Infant weight	0.70 (0.67–0.72)	< 0.001	0.66 (0.61–0.71)	< 0.001
Second stage of labor	0.76 (0.74–0.78)	< 0.001	0.73 (0.68–0.77)	< 0.001
Forceps delivery	0.52 (0.51–0.54)	< 0.001	0.55 (0.52–0.59)	< 0.001

C-statistic, concordance statistic; CI, confidence interval

*Delong test were used for comparing C-statistic

## Discussion

In this study, we developed and validated a simple nomogram to quantify the risks of developing SUI in the early postpartum period. This nomogram, based on maternal and obstetrical characteristics, had excellent discriminatory ability, calibration, and net benefit in predicting SUI. Early rehabilitation is of critical importance for prevention of progression and persistence of SUI [25, 26]. Thus, early prediction of SUI could help clinicians provide professional counseling as well as rehabilitation guidance to the appropriate population.

A prediction model has previously been developed to assess the probability of early postpartum SUI among 360 primiparous women [2]. However, the efficacy of the model is limited by the small sample size, the combined study property of cesarean and vaginal delivery, and the lack of validation. Pregnancy and delivery are most important risk factors of SUI [27, 28], and there were significant differences in baseline factors between cesarean and vaginal deliveries. In the present study, we created a nomogram in a larger cohort based on a detailed collection of obstetrical and especially labor-related factors, which have been well validated both internally and externally. To our knowledge, this is the first nomogram to collect detailed delivery information for predicting the occurrence of postpartum SUI among women with vaginal deliveries. Five factors were identified to be predictive of postpartum SUI in the model, namely age, parity, the duration of second stage of labor, forceps delivery, and infant weight.

SUI was generally considered to be strongly associated with maternal age. As age increases, the contractility of pelvic floor muscle fibers and fascial tone decreases, which may lead to poor stability of the pelvic floor structures. Chang et al. has increased prevalence of SUI in women aged ≥ 30 years during the postpartum period [29]. In addition, Chuang et.al found a significant association between age and SUI when age was treated as a continuous variable [30]. Similarly, we found that the risk of postpartum SUI increased with each additional year of age. Therefore, more attention should be paid to aging maternity regarding the occurrence of the postpartum SUI.

We found that parity was a valuable predictor for SUI, which is consistent with previous studies [29, 31]. On one hand, high levels of hormone exposure in pregnancy, especially estradiol, can adversely affect metabolism of pelvic floor muscle fibers [32]. On the other hand, it seems that mechanical injuries to the pelvic floor structures during vaginal delivery have cumulative effects with an increasing number of deliveries. Prolonged second stage of labor was shown to be another important predictor of postpartum SUI by our study and previous studies [33–35]. The anatomical support of the bladder neck and the urethra may be lessened if damage to the arcus tendineus fasciae pelvis or paravaginal tissue occurs as a result of excessive loading from the continuously descending fetal head [27]. Therefore, it indicates that both the increased number of impairments and the prolonged duration of stress on the pelvic floor structure are associated with the onset of postpartum SUI. Birthweight of infant has been found to be associated with a risk of incontinence [36, 37]. After correction for confounding factors, there was no association between infant weight and postnatal SUI in our study, which may result from the combined effects of study population differences, hospital condition, and postpartum screening methods. However, infant weight showed good performance in stepwise regression, suggesting the prolonged pressure induced by high birthweight on the pelvic floor might increase the risk of SUI to some extent.

So far, the risk of SUI for forceps delivery, vacuum delivery, and spontaneous vaginal birth has not been evaluated in randomized trials. Two longitudinal studies have investigated the association between SUI and delivery mode. In the first study [38], among 1,528 different race women who were followed up by the investigators through questionnaires for up to 9 years, no difference was found in the cumulative incidence of SUI by mode of delivery. However, participants with forceps and vacuum delivery in this study were analyzed mixedly as operative vaginal birth, limiting the power. In the second study [39], 13,694 women from Norway completed questionnaires many years after delivery. Forceps delivery was associated with a higher risk of SUI with an OR of 1.42 (95% CI 1.09–1.86) and an OR of 1.76 (95% CI 1.19–2.60) compared with spontaneous vaginal delivery, and vacuum delivery respectively. Our findings were identical to the latter, but the increased risk of SUI associated with the use of forceps was more significant with an OR of 6.08 (95% CI 3.85–9.56). This discrepancy may be due to differences in the timing and types of forceps, which remain to be further investigated.

The current study has several limitations. First, SUI symptoms were self-reported, and there were no objective measurements for determining SUI such as a cough stress test, a pad test, or urodynamic testing, so they were subject to recall bias. Second, it is well known that C-index decreases with an increase in follow up duration. Thus, the study presents the limitation of having a short postpartum duration and follow up duration for determining risk of SUI. Also, we have only determined the incidence of SUI and not its duration or severity. Third, some potential factors regarding demographics and the postpartum recovery phase (e.g., economic level, postpartum breastfeeding, whether pelvic floor function exercise, etc.) have not been taken into account. More potential indicators combined with clinical characteristics are warranted to be investigated to build a more accurate prediction model for postpartum SUI. Fourth, the current study is a retrospective study, which has an inherent selection bias and some important variables, such as breastfeeding, that cannot be collected. Finally, almost all the participants in this study were residents of Shanghai, an economically developed region of China. The model has not been externally validated by multicenter data. Therefore, our results cannot be extrapolated to all populations.

The novel nomogram in the present study has practice implications due to the fact that it is simple to adopt, shows well discrimination, and demonstrates good calibration to predict the occurrence of postpartum SUI among women with vaginal delivery. The nomogram might help women with vaginal delivery benefit from early detection and rehabilitation as with SUI, which will facilitate the management of pelvic floor disorders. However, the benefits remain to be explored in prospective trials.

## Conclusion

We have created a nomogram that can be utilized to quantify the risk of postpartum SUI for women with vaginal delivery. The model might contribute to early identification of postpartum SUI, thereby facilitating the management of SUI.

## Supplementary Information

**Additional file 1**. Supplementary Table.

## Data Availability

The data analyzed during the study is available from the corresponding author upon reasonable request.

## Abbreviations

SUI: Stress urinary incontinence
C-statistics: Concordance statistics
CI: Confidence interval
BMI: Body mass index
EMR: Electronic medical record
HER: Electronic health record
IUGA: International Urogynecological Association
ICS: International Continence Society
ROC: Receiver operating characteristic
OR: Odds ratio

## References

[1] Haylen BT, de Ridder D, Freeman RM, Swift SE, Berghmans B, Lee J, Monga A, Petri E, Rizk DE, Sand PK (2010). An International Urogynecological Association (IUGA)/International Continence Society (ICS) joint report on the terminology for female pelvic floor dysfunction. Neurourol Urodyn.

[2] Cheng H, Gong F, Shen Y, OuYang P, Ni R, Gao H (2022). A nomogram model predicting the risk of postpartum stress urinary incontinence in primiparas: a multicenter study. Taiwan J Obstet Gynecol.

[3] Dinc A (2018). Prevalence of urinary incontinence during pregnancy and associated risk factors. Low Urin Tract Symptoms.

[4] Arrue M, Ibanez L, Paredes J, Murgiondo A, Belar M, Sarasqueta C, Diez-Itza I (2010). Stress urinary incontinence six months after first vaginal delivery. Eur J Obstet Gynecol Reprod Biol.

[5] MacArthur C, Wilson D, Herbison P, Lancashire RJ, Hagen S, Toozs-Hobson P, Dean N, Glazener C (2016). Prolong study g: Urinary incontinence persisting after childbirth: extent, delivery history, and effects in a 12-year longitudinal cohort study. BJOG Int J Obstet Gynaecol.

[6] MacArthur C, Glazener CM, Wilson PD, Lancashire RJ, Herbison GP, Grant AM (2006). Persistent urinary incontinence and delivery mode history: a six-year longitudinal study. BJOG Int J Obstet Gynaecol.

[7] Monti M, Fischetti M, Santangelo G, Galli V, Clemente F, Giannini A, Tibaldi V, Pecorini F, Perniola G (2021). Urinary incontinence in women: state of the art and medical treatment. Minerva Obstet Gynecol.

[8] Monti M, Fischetti M, Di Pinto A, Santangelo G, Giannini A, D'Oria O, Golia D'Augè T, Carbone F, Perniola G (2021). Update on surgical treatment of female stress urinary incontinence. Minerva Obstet Gynecol.

[9] Subak LL, Brown JS, Kraus SR, Brubaker L, Lin F, Richter HE, Bradley CS, Grady D (2006). Diagnostic aspects of incontinence study G: the “costs” of urinary incontinence for women. Obstet Gynecol.

[10] Wood LN, Anger JT (2014). Urinary incontinence in women. BMJ (Clin Res Ed).

[11] Brown SJ, Donath S, MacArthur C, McDonald EA, Krastev AH (2010). Urinary incontinence in nulliparous women before and during pregnancy: prevalence, incidence, and associated risk factors. Int Urogynecol J.

[12] Nygaard I, Barber MD, Burgio KL, Kenton K, Meikle S, Schaffer J, Spino C, Whitehead WE, Wu J, Brody DJ (2008). Prevalence of symptomatic pelvic floor disorders in US women. JAMA.

[13] Rortveit G, Daltveit AK, Hannestad YS, Hunskaar S, Norwegian ES (2003). Urinary incontinence after vaginal delivery or cesarean section. N Engl J Med.

[14] Tahtinen RM, Cartwright R, Tsui JF, Aaltonen RL, Aoki Y, Cardenas JL, El Dib R, Joronen KM, Al Juaid S, Kalantan S (2016). Long-term impact of mode of delivery on stress urinary incontinence and urgency urinary incontinence: a systematic review and meta-analysis. Eur Urol.

[15] Tahtinen RM, Cartwright R, Vernooij RWM, Rortveit G, Hunskaar S, Guyatt GH, Tikkinen KAO (2019). Long-term risks of stress and urgency urinary incontinence after different vaginal delivery modes. Am J Obstet Gynecol.

[16] Hunskaar S (2008). A systematic review of overweight and obesity as risk factors and targets for clinical intervention for urinary incontinence in women. Neurourol Urodyn.

[17] Rortveit G, Hunskaar S (2006). Urinary incontinence and age at the first and last delivery: the Norwegian HUNT/EPINCONT study. Am J Obstet Gynecol.

[18] Gyhagen M, Bullarbo M, Nielsen TF, Milsom I (2013). A comparison of the long-term consequences of vaginal delivery versus caesarean section on the prevalence, severity and bothersomeness of urinary incontinence subtypes: a national cohort study in primiparous women. BJOG Int J Obstet Gynaecol.

[19] Connolly TJ, Litman HJ, Tennstedt SL, Link CL, McKinlay JB (2007). The effect of mode of delivery, parity, and birth weight on risk of urinary incontinence. Int Urogynecol J Pelvic Floor Dysfunct.

[20] Thom DH, Brown JS, Schembri M, Ragins AI, Creasman JM, Van Den Eeden SK (2011). Parturition events and risk of urinary incontinence in later life. Neurourol Urodyn.

[21] Gyhagen M, Bullarbo M, Nielsen TF, Milsom I (2013). The prevalence of urinary incontinence 20 years after childbirth: a national cohort study in singleton primiparae after vaginal or caesarean delivery. BJOG Int J Obstet Gynaecol.

[22] Diez-Itza I, Arrue M, Ibanez L, Murgiondo A, Paredes J, Sarasqueta C (2010). Factors involved in stress urinary incontinence 1 year after first delivery. Int Urogynecol J.

[23] Mikuš M, Ćorić M, Matak L, Škegro B, Vujić G, Banović V (2020). Validation of the UDI-6 and the ICIQ-UI SF-Croatian version. Int Urogynecol J.

[24] Huang L, Zhang SW, Wu SL, Ma L, Deng XH (2008). The Chinese version of ICIQ: a useful tool in clinical practice and research on urinary incontinence. Neurourol Urodyn.

[25] Mørkved S, Bø K (2014). Effect of pelvic floor muscle training during pregnancy and after childbirth on prevention and treatment of urinary incontinence: a systematic review. Br J Sports Med.

[26] Woodley SJ, Boyle R, Cody JD, Mørkved S, Hay-Smith EJC (2017). Pelvic floor muscle training for prevention and treatment of urinary and faecal incontinence in antenatal and postnatal women. Cochrane Database Syst Rev.

[27] Aoki Y, Brown HW, Brubaker L, Cornu JN, Daly JO, Cartwright R (2017). Urinary incontinence in women. Nat Rev Dis Primers.

[28] Hu JS, Pierre EF (2019). Urinary incontinence in women: evaluation and management. Am Fam Physician.

[29] Chang SR, Lin WA, Chang TC, Lin HH, Lee CN, Lin MI (2021). Risk factors for stress and urge urinary incontinence during pregnancy and the first year postpartum: a prospective longitudinal study. Int Urogynecol J.

[30] Chuang CM, Lin IF, Horng HC, Hsiao YH, Shyu IL, Chou P (2012). The impact of gestational diabetes mellitus on postpartum urinary incontinence: a longitudinal cohort study on singleton pregnancies. BJOG.

[31] Chang SR, Lin WA, Lin HH, Lee CN, Chang TC, Lin MI (2022). Cumulative incidence of urinary incontinence and associated factors during pregnancy and after childbirth: a cohort study. Int Urogynecol J.

[32] Bodner-Adler B, Bodner K, Kimberger O, Halpern K, Rieken M, Koelbl H, Umek W (2017). Role of serum steroid hormones in women with stress urinary incontinence: a case–control study. BJU Int.

[33] Wang K, Xu X, Jia G, Jiang H (2020). Risk factors for postpartum stress urinary incontinence: a systematic review and meta-analysis. Reprod Sci (Thousand Oaks, Calif).

[34] Wang Q, Yu X, Sun X, Wang J (2020). Does epidural anesthesia influence pelvic floor muscle endurance and strength and the prevalence of urinary incontinence 6 weeks postpartum?. Int Urogynecol J.

[35] Guo KM, He LC, Feng Y, Huang L, Morse AN, Liu HS (2021). Surface electromyography of the pelvic floor at 6–8 weeks following delivery: a comparison of different modes of delivery. Int Urogynecol J.

[36] Daly D, Clarke M, Begley C (2018). Urinary incontinence in nulliparous women before and during pregnancy: prevalence, incidence, type, and risk factors. Int Urogynecol J.

[37] Arrue Gabilondo M, Ginto L, Zubikarai M, Galán C, Saro J, Diez-Itza I (2021). Risk factors associated with stress urinary incontinence 12 years after first delivery. Int Urogynecol J.

[38] Blomquist JL, Muñoz A, Carroll M, Handa VL (2018). Association of delivery mode with pelvic floor disorders after childbirth. JAMA.

[39] Tähtinen RM, Cartwright R, Vernooij RWM, Rortveit G, Hunskaar S, Guyatt GH, Tikkinen KAO (2019). Long-term risks of stress and urgency urinary incontinence after different vaginal delivery modes. Am J Obstet Gynecol.

