# Supplementary material for: Establishment and validation of a simple nomogram for predicting early postpartum stress urinary incontinence among women with vaginal delivery: a retrospective study
Source: BMC Womens Health. 2023 Jan 9;23:8. doi: 10.1186/s12905-023-02160-2 (PMC9827703; doi:10.1186/s12905-023-02160-2)
Supplement: Supplementary file 1 — Additional file 1. Supplementary Table. [file 12905_2023_2160_MOESM1_ESM.docx]

sTable 1 Comparison of baseline data for excluded and included populations.

| Characteristics | Excluded group  （N=3982） | Included group  （N=3051） | P value |
| --- | --- | --- | --- |
| Age, y | 31 (29, 33) | 31.00 (29, 33) | 0.928 |
| Parity |  |  | 0.662 |
| 1 | 2440 (61.3) | 1853 (60.7) |  |
| ≥ 2 | 1542 (38.7) | 1198 (39.3) |  |
| Pre-pregnancy BMI, kg/m2 | 22.4 (19.8, 26.3) | 22.3 (19.8, 26.2) | 0.873 |

Abbreviations: BMI, body max index
